# Getting started with tables

**DOI:** 10.1186/s13690-017-0180-1

**Published:** 2017-03-20

**Authors:** Hazel Inskip, Georgia Ntani, Leo Westbury, Chiara Di Gravio, Stefania D’Angelo, Camille Parsons, Janis Baird

**Affiliations:** MRC Lifecourse Epidemiology Unit, University of Southampton, Southampton General Hospital, Southampton, SO16 6YD UK

**Keywords:** Tables, Variables, Characteristics, Categories, Mean, Standard deviation, Median, Inter-quartile range, Regression coefficients, Correlation coefficients, Ratios, Relative measures

## Abstract

**Background:**

Tables are often overlooked by many readers of papers who tend to focus on the text. Good tables tell much of the story of a paper and give a richer insight into the details of the study participants and the main research findings. Being confident in reading tables and constructing clear tables are important skills for researchers to master.

**Method:**

Common forms of tables were considered, along with the standard statistics used in them. Papers in the Archives of Public Health published during 2015 and 2016 were hand-searched for examples to illustrate the points being made. Presentation of graphs and figures were not considered as they are outside the scope of the paper.

**Results:**

Basic statistical concepts are outlined to aid understanding of each of the tables presented. The first table in many papers gives an overview of the study population and its characteristics, usually giving numbers and percentages of the study population in different categories (e.g. by sex, educational attainment, smoking status) and summaries of measured characteristics (continuous variables) of the participants (e.g. age, height, body mass index). Tables giving the results of the analyses follow; these often include summaries of characteristics in different groups of participants, as well as relationships between the outcome under study and the exposure of interest. For continuous outcome data, results are often expressed as differences between means, or regression or correlation coefficients. Ratio/relative measures (e.g. relative risks, odds ratios) are usually used for binary outcome measures that take one of two values for each study participants (e.g. dead versus alive, obese versus non-obese). Tables come in many forms, but various standard types are described here.

**Conclusion:**

Clear tables provide much of the important detail in a paper and researchers are encouraged to read and construct them with care.

## Background

Tables are an important component of any research paper. Yet, anecdotally, many people say that they find tables difficult to understand so focus only on the text when reading a paper. However, tables provide a much richer sense of a study population and the results than can be described in the text. The tables and text complement each other in that the text outlines the main findings, while the detail is contained in the tables; the text should refer to each table at the appropriate place(s) in the paper. We aim to give some insights into reading tables for those who find them challenging, and to assist those preparing tables in deciding what they need to put into them. Producing clear, informative tables increases the likelihood of papers being published and read. Good graphs and figures can often provide a more accessible presentation of study findings than tables. They can add to the understanding of the findings considerably, but they can rarely contain as much detail as a table. Choosing when to present a graph or figure and when to present a table needs careful consideration but this article focuses only on the presentation of tables.

We provide a general description of tables and statistics commonly used when presenting data, followed by specific examples. No two papers will present the tables in the same way, so we can only give some general insights. The statistical approaches are described briefly but cannot be explained fully; the reader is referred to various books on the topic [1–6].

### Presentation of tables

The title (or legend) of a table should enable the reader to understand its content, so a clear, concise description of the contents of the table is required. The specific details needed for the title will vary according to the type of table. For example, titles for tables of characteristics should give details of the study population being summarised and indicate whether separate columns are presented for particular characteristics, such as sex. For tables of main findings, the title should include the details of the type of statistics presented or the analytical method. Ideally the table title should enable the table to be examined and understood without reference to the rest of the article, and so information on study, time and place needs to be included. Footnotes may be required to amplify particular points, but should be kept to a minimum. Often they will be used to explain abbreviations or symbols used in the table or to list confounding factors for which adjustment has been made in the analysis.

Clear headings for rows and columns are also required and the format of the table needs careful consideration, not least in regard to the appropriateness and number of rows and columns included within the table. Generally it is better to present tables with more rows than columns; it is usually easier to read down a table than across it, and page sizes currently in use are longer than they are wide. Very large tables can be hard to absorb and make the reader’s work more onerous, but can be useful for those who require extra detail. Getting the balance right needs care.

### Types of tables

Many research articles present a summary of the characteristics of the study population in the first table. The purpose of these tables is to provide information on the key characteristics of the study participants, and allow the reader to assess the generalisability of the findings. Typically, age and sex will be presented along with various characteristics pertinent to the study in question, for example smoking prevalence, socio-economic position, educational attainment, height, and body mass index. A single summary column may be presented or perhaps more than one column split according to major characteristics such as sex (i.e. separate columns for males and females) or, for trials, the intervention and control groups.

Subsequent tables generally present details of the associations identified in the main analyses. Sometimes these include results that are unadjusted or ‘crude’ (i.e. don’t take account of other variables that might influence the association) often followed by results from adjusted models taking account of other factors.

Other types of tables occur in some papers. For example, systematic review papers contain tables giving the inclusion and exclusion criteria for the review as well as tables that summarise the characteristics and results of each study included in the review; such tables can be extremely large if the review covers many studies. Qualitative studies often provide tables describing the characteristics of the study participants in a more narrative format than is used for quantitative studies. This paper however, focuses on tables that present numerical data.

### Statistics commonly presented in tables

**Fig. 1:**
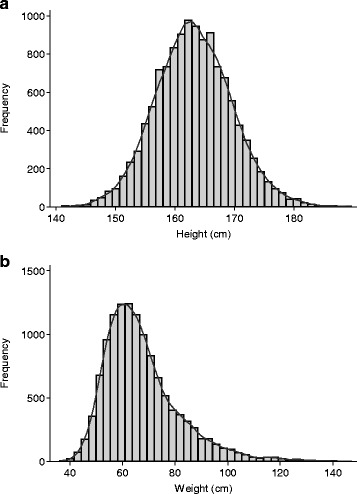
Distribution of heights and weights of young women from the Southampton Women’s Survey [7]. **a** Shows the height distribution, which is symmetrical and generally follows a standard normal distribution, while **b** shows weight, which is skewed to the right

The main summary statistics provided within a table depend on the type of outcome under investigation in the study. If the variable is continuous (i.e. can take any numerical value, between a minimum and a maximum, such as blood pressure, height, birth weight), then means and standard deviations (SD) tend to be given when the distribution is symmetrical, and particularly when it follows the classical bell shaped curve known as a Normal or Gaussian distribution (see Fig. 1a). The mean is the usual arithmetic average and the SD is an indication of the spread of the values. Roughly speaking, the SD is about a quarter of the difference between the largest and the smallest value excluding 5% of values at the extreme ends. So, if the mean is 100 and the SD is 20 we would expect 95% of the values in our data to be between about 60 (i.e. 100–2×20) and 140 (100 + 2×40).

The median and inter-quartile range (IQR) are usually provided when the data are not symmetrical as in Fig. 1b, which gives an example of data that are skewed, such that if the values are plotted in a histogram there are many values at one end of the distribution but fewer at the other end [7]. If all the values of the variable were listed in order, the median would be the middle value and the IQR would be the values a quarter and three-quarters of the way through the list. Sometimes the lower value of the IQR is labelled Q1 (quartile 1), the median is Q2, and the upper value is Q3. For categorical variables, frequencies and percentages are used.

Common statistics for associations between continuous outcomes include differences in means, regression coefficients and correlation coefficients. For these statistics, values of zero indicate no association between the exposure and outcome of interest. A correlation coefficient of 0 indicates no association, while a value of 1 or −1 would indicate perfect positive or negative correlation; values outside the range −1 to 1 are not possible. Regression coefficients can take any positive or negative value depending on the units of measurement of the exposure and outcome.

For binary outcome measures that only take two possible values (e.g. diseased versus not, dead versus alive, obese versus not obese) the results are commonly presented in the form of relative measures. These include any measure with the word ‘relative’ or ‘ratio’ in their name, such as odds ratios, relative risks, prevalence ratios, incidence rate ratios and hazard ratios. All are interpreted in much the same way: values above 1 indicate an elevated risk of the outcome associated with the exposure under study, whereas below 1 implies a protective effect. No association between the outcome and exposure is apparent if the ratio is 1.

Typically in results tables, 95% confidence intervals (95% CIs) and/or *p*-values will be presented. A 95% CI around a result indicates that, in the absence of bias, there is a 95% probability that the interval includes the true value of the result in the wider population from which the study participants were drawn. It also gives an indication of how precisely the study team has been able to estimate the result (whether it is a regression coefficient, a ratio/relative measure or any of the summary measures mentioned above). The wider the 95% CI, the less precise is our estimate of the result. Wide 95% CIs tend to arise from small studies and hence the drive for larger studies to give greater precision and certainty about the findings.

If a 95% CI around a result for a continuous variable (difference in means, regression or correlation coefficient) includes 0 then it is unlikely that there is a real association between exposure and outcome whereas, for a binary outcome, a real association is unlikely if the 95% CI around a relative measure, such as a hazard or odds ratio, includes 1.

The *p*-value is the probability that the finding we have observed could have occurred by chance, and therefore there is no identifiable association between the exposure of interest and the outcome measure in the wider population. If the *p*-value is very small, then we are more convinced that we have found an association that is not explained by chance (though it may be due to bias or confounding in our study). Traditionally a *p*-value of less than 0.05 (sometimes expressed as 5%) has been considered as ‘statistically significant’ but this is an arbitrary value and the smaller the *p*-value the less likely the result is simply due to chance [8].

Frequently, data within tables are presented with 95% CIs but without *p*-values or vice versa. If the 95% CI includes 0 (for a continuous outcome measure) or 1 (for a binary outcome), then generally the *p*-value will be greater than 0.05, whereas if it does not include 0 or 1 respectively, then the *p*-value will be less than 0.05 [9]. Generally, 95% CIs are more informative than *p*-values; providing both may affect the readability of a table and so preference should generally be given to 95% CIs. Sometimes, rather than giving exact p-values, they are indicated by symbols that are explained in a footnote; commonly one star (*) indicates *p* < 0.05, two stars (**) indicates *p* < 0.01.

Results in tables can only be interpreted if the units of measurement are clearly given. For example, mean or median age could be in days, weeks, months or years if infants and children are being considered, and 365, 52, 12 or 1 for a mean age of 1 year could all be presented, as long the unit of measurement is provided. Standard deviations should be quoted in the same units as the mean to which they refer. Relative measures, such as odds ratios, and correlation coefficients do not have units of measurement, but for regression coefficients the unit of measurement of the outcome variable is required, and also of the exposure variable if it is continuous.

### Examples

The examples are all drawn from recent articles in Archives of Public Health. They were chosen to represent a variety of types of tables seen in research publications.

### Tables of characteristics

The table of characteristics in Table 1 is from a study assessing knowledge and practice in relation to tuberculosis control among in Ethiopian health workers [10]. The authors have presented the characteristics of the health workers who participated in the study. Summary statistics are based on categories of the characteristics, so numbers (frequencies) in each category and the percentages of the total study population within each category are presented for each characteristic. From this, the reader can see that:

- the study population is quite young, as only around 10% are more than 40 years old;
- the majority are female;
- more than half are nurses;
- about half were educated to degree level or above.

**Table 1:** Table of study population characteristics from a paper on the assessment of knowledge and practice in relation to tuberculosis control in health workers in Ethiopia [10]. Socio demographic characteristics of the study population in public health facilities, Addis Ababa, 2014

Variable	Characteristics	Frequency	Percent
		(*N*=582)	
Age	18–29	383	65.8
	30–39	136	23.4
	>40	63	10.4
Sex	Male	228	39.2
	Female	352	60.5
Marital status	Single	308	52.9
	Married	260	44.7
	Divorced and Widowed	14	2.4
Profession	Physician	35	6
	Nurse	66	56.4
	Health Officer	328	11.3
	Lab personnsel	49	8.4
	Pharmacy personnsel	45	7.7
	Others^a^	59	10.1
Currently working unit	OPD	181	31.1
	TB clinic and TB ward	30	5.2
	Laboratory	43	7.4
	Pharmacy	46	7.9
	Triage	24	4.1
	Medical ward	32	5.5
	Others^b^	226	38.8
Educational status	Diploma	280	48.1
	First degree	289	49.7
	Second degree and above	13	2.2
Service year in health facility	<3 years	341	58.6
	3-6 year	150	25.8
	>6 years	91	15.6
Experience in TB clinics	Yes	134	23
	No	444	76.3
Year of experience in TB clinic	<1 year	57	57
	1-4 years	37	37
	>4 years	6	6
Have TB training	Yes	134	23
	No	444	76.3
Duration of training	<3 days	23	17.6
	4-6 days	59	45
	7-10 days	35	28.2
	>10 days	12	9.2

*OPD* outpatient department; *TB* Tuberculosis.

^a^Midwife, radiology, physiotherapy; ^b^MCH, delivery,EPI, FP, physiotherapy

The table of characteristics in Table 2 is from a study of the relationship between distorted body image and lifestyle in adolescents in Japan [11]. Here the presentation is split into separate columns for boys and girls. The first four characteristics are continuous variables, not split into categories but, instead, presented as means, with the SDs given in brackets. The three characteristics in the lower part of the table are categorical variables and, similar to Table 1, the frequency/numbers and percentages in each category are presented. The *p*-values indicate that boys and girls differ on some of the characteristics, notably height, self-perceived weight status and body image perception.

**Table 2:** Table of study population characteristics from a paper on the relationship between distorted body image and lifestyle in adolescents in Japan [11]. Characteristics of study participants by sex (Japan; 2005–2009)

Variable	Boys	Girls	*P*-value
	(*n*=885)	(*n*=846)	
Age (years)	12.3 (0.4)	12.3 (0.4)	0.631
Height (cm)	154.4 (8.1)	152.5 (6.0)	<0.001
Weight (kg)	44.5(9.7)	43.6 (7.9)	0.040
Body mass index (kg/m^2)^	18.5 (3.0)	1837 (2.7)	0.276
Actual weight (%)			
Underweight	73 (8.2)	88 (10.4)	0.116
Normal weight	694 (78.4)	666 (78.7)	
Overweight	118 (13.3)	92 (10.9)	
Self-perceived weight status (%)			
Thin	268 (30.3)	139 (16.4)	<0.001
Normal	484 (54.7)	560 (59.8)	
Heavy	133 (15.0)	201 (23.8)	
Body image perception (%)			
Underestimated	230 (26.0)	99 (11.7)	<0.001
Correct	605 (68.4)	591 (69.9)	
Overestimated	50 (5.6)	156 (18.4)	

Data are expressed as numbers (%), values are means (standard deviation). The unpaired *t-*test and chi-squad test were used to compare characteristics between boys and girls

In Table 3, considerable detail is given for continuous variables in the table. This comes from an article describing the relationship between mid-upper-arm circumference (MUAC) and weight changes in young children admitted to hospital with severe acute malnutrition from three countries [12]. For each country, the categorical characteristic of sex is presented as in the previous two examples, but more detail is given for the continuous variables of age, MUAC and height. The mean is provided as in Table 2, though without a standard deviation, but we are also given the minimum value, the 25th percentile (labelled Q1 – for quartile 1), the median (the middle value), the 75th percentile (labelled Q2, here though correctly it should be Q3 – see above) and the maximum value. The table shows:

- Ethiopian children in this study were older and taller than those from the other two countries but their MUAC measurements tended to be smaller;
- in Bangladesh, disproportionally more females than males were admitted for treatment compared with the other two countries.

**Table 3:** Table of study population characteristics from a paper describing the relationship between mid-upper-arm circumference (MUAC) and weight changes in young children [12]. Characteristics of study population at admission

Ethiopia		n	%				
	Males	199	46.2%				
	Females	232	53.8%				
		Min.	Q1	Median	Mean	Q2	Max.
	Age at admission (months)	7.0	25.1	37.0	39.5	48.0	66.0
	MUAC at admission (cm)	7.5	10.2	10.5	10.4	10.8	10.9
	Height at admission (cm)	61.5	73.5	80.4	81.0	88.0	109.2
Malawi		n	%				
	Males	105	44.7%				
	Females	130	55.3%				
		Min.	Q1	Median	Mean	Q2	Max
	Age at admission (months)	6.0	10.0	14.0	16.4	21.0	51.0
	MUAC at admission (cm)	8.2	10.5	11.0	10.8	11.4	11.5
	Height at admission (cm)	53.3	63.0	67.2	67.5	72.2	92.5
Bangladesh		n	%				
	Males	88	33.3%				
	Females	176	66.7%				
		Min.	Q1	Median	Mean	Q2	Max.
	Age at admission (months)	6.0	7.0	10.0	12.9	17.0	56.0
	MUAC at admission (cm)	8.5	11.1	11.3	11.2	11.4	11.4
	Height at admission (cm)	51.6	62.3	65.6	67.4	71.8	99.0

It is unusual to present as much detail on continuous characteristics as is given in Table 3 . Usually, for each characteristic, either (a) mean and SD or (b) median and IQR would be given, but not both.

### Tables of results – summary findings

Many results tables are simple summaries and look similar to tables presenting characteristics, as described above. Sometimes the initial table of characteristics includes some basic comparisons that indicate the main results of the study. Table 4 shows part of a large table of characteristics for a study of risk factors for acute lower respiratory infections (ALRI) among young children in Rwanda [13]. In addition to presenting the numbers of children in each category of a variety of characteristics, it also shows the percentage in each category among those who suffered ALRI in the previous two weeks, and provides *p-*values for the differences between the categories among those who did and did not suffer from ALRI. Thus only 2.9% of older children (24–59 months) within the study suffered from ALRI, compared with about 5% in the two youngest categories. The *p*-value of 0.001, well below 0.05, indicates that this difference is statistically significant. The other finding of some interest is that children who took vitamin A supplements appeared to be less likely to suffer from ALRI than those who did not, but the *p*-value of 0.04 is close to 0.05 so not as remarkable a finding as for the difference between the age groups.

**Table 4:** Part of a table of basic results from a study of risk factors for acute lower respiratory infections (ALRI) among young children in Rwanda [13]. Bivariate analysis of factors associated with acute lower respiratory infection among children under five in Rwanda, RDHS 2010

Name of Variable	Children in study Number	Children suffering fronALRI in last two weeks Number (%)	Chi-squared *p*-value
CHILD			0.001
Child age		82 (5.2)	
0-11 months	1,573		
12-23 months	1,615	82 (5.1)	
24-59 months	5,411	157 (2.9)	
Child sex			0.104
Boy	4,361	179 (4.1)	
Girl	4,238	144 (3.4)	
Child underweight			0.991
No	3,648	139 (3.8)	
Yes	467	18 (3.8)	
Not measured	4,424	164 (3.7)	
Child received BCG			0.109
No	94	1 (0.9)	
Yes	8,503	323 (3.8)	
Child received intestinal drugs in last 6 months			0.119
No	94	4 (4.4)	
Yes	8,503	306 (3.6)	
Anemia level			0.083
Not anemic	2,316	74 (3.2)	
Mild or moderate	1,441	60 (4.2)	
Severe	17	2 (14.6)	
Not measured	4,424	164 (3.7)	
Child received vitamin A in last 6 months			0.040
No	1,109	54 (4.9)	
Yes	7,484	269 (3.6)	
Child delivered at a health facility			0.326
No	2,625	89 (3.4)	
Yes	5,969	233 (3.9)	
PARENT			
Mother current age			0.178
<21 years	273	14 (5.3)	
21+ years	8,326	308 (3.7)	
Mother employment status			0.225
Not working or self-employed agriculture	7,488	269 (3.6)	
Working	1,100	50 (4.6)	
Mother education level			0.210
Less than secondary	7,837	282 (3.6)	
Secondary or high	762	37 (4.9)	
Partner education level			0.406
Less than secondary	7,155	257 (3.6)	
Secondary or higher	882	40 (4.4)	

Table 5 shows a summary table of average life expectancy in British Columbia by socioeconomic status [14]. The average life expectancy at birth and the associated 95% CIs are given according to level of socio-economic status for the total population (column 1), followed by males and females separately. The study is large so the 95% CIs are quite narrow, and the table indicates that there are considerable differences in life expectancy between the three socioeconomic groups, with the lowest category having the poorest life expectancy. The gap in life expectancy between the lowest and highest category is more than three years, as shown in the final row.

**Table 5:** Summary table of average life expectancy in British Columbia by socioeconomic status [14]. British Columbia regional average life expectancy at birth by regional socioeconomic status, 2007–2011

SES category	Total LE_0_ (95% CI)	Male LE_0_ (95% CI)	Female LE_0_ (95% CI)
Low	78.6 (78.0-79.3)	76.6 (75.7-77.5)	81.1 (80.4-81.8)
Medium	80.5 (79.8-81.1)	78.2 (77.5-78.9)	82.8 (82.0-83.5)
High	82.2 (81.6-82.8)	80.2 (79.5-81.0)	84.2 (83.7-84.8)
LE_0_ Gap between low and high SES	3.6	3.6	3.1

*SES* Socioeconomic status, *LE*_0_ Life expectancy at birth, *CI* Confidence interval

### Tables of results – continuous outcomes

Continuous outcome measures can be analysed in a variety of ways, depending on the purpose of the study and whether the measure of the exposure is continuous, categorical or binary.

Table 6 shows an example of correlation coefficients indicating the degree of association between the exposure of interest (cognitive test scores) and the outcome measure (academic performance) [15]. No confidence intervals are presented, but the results show that almost all the particular cognitive test scores are statistically significantly associated (*p*-value < 0.05) with the two measures of academic performance. Note that this table is an example of where a footnote is used to give information about the p-values. Not surprisingly, all the correlations are positive; one would expect that as cognitive score increase so too would academic performance. The numbers labelled “N” give the number of children who contributed data to each correlation coefficient.

**Table 6:** Correlation coefficients from a study assessing the association between cognitive function and academic performance in Ethiopia [15]. Correlation between cognitive fuinction test and academic performance among school aged children in Goba Town, South east Ethiopia, May 2014

Cognitive test scores		Academic performance	
		Average semester result	Mathematics
Number Recall score	r	0.14	0.19*
	*p*-value	0.12	0.03
	N	131	130
Rovers score	r	0.22*	0.22*
	*p*-value	.013	0.01
	N	131	130
Hand Movement score	r	0.16	0.20*
	*p*-value	0.08	0.03
	N	131	130
Pattern score	r	0.24**	0.27**
	*P*-value	0.005	0.002
	N	131	130
Word Order score	r	0.23**	0.19*
	*p*-value	0.008	0.028
	N	131	130
Triangles test score	r	0.33**	0.29**
	*p*-value	0.001	0.001
	N	131	130
Raven CPMtest score	r	0.38**	0.38**
	*p*-value	0.001	<0.001
	N	129	128

*Statistically significant at *p*<0.05, **Statistically significant a *p*>0.01

Table 7 is quite a complex table, but one that bears examination. It presents regression coefficients from an analysis of pregnancy exposure to nitrogen dioxide (NO_2_) and birth weight of the baby in a large study of four areas in Norway; more than 17,000 women-baby pairs contributed to the complete crude analysis [16]. Regression coefficients are presented and labelled “Beta”, the usual name for such coefficients, though the Greek letter β, B or b are sometimes used. They are interpreted as follows: for one unit increase in the exposure variable then the outcome measure increases by the amount of the regression coefficient. Regression coefficients of zero indicate no association. In this table, the Beta in the top left of the table indicates that as NO_2_ exposure of the mother increases by 1 unit (a ‘unit’ in this analysis is 10 μg/m^3^, see the footnote in the table, which gives the units of measurement used for the regression coefficients: grams per 10 μg/m^3^ NO_2_) then the birth weight of her baby decreases (because the Beta is negative) by 37.9 g. The 95% CI does not include zero and the *p*-value is small (<0.001) implying that the association is not due solely to chance.

**Table 7:** Table of regression coefficients for the relationship between exposure to NO_2_ in pregnancy and birth weight [16]. Main and stratified analysis of association between pregnancy exposure to NO_2_ and birth weight

	Crude			Model 1^a^			Model 2^b^			Model 3+c		
	N	Beta 95% CI	*p*-value	N	Beta 95% CI	*p*-value	N	Beta 95% CI	*p*-value	N	Beta 95% CI	*p*-value
Main analysis												
Entire study population	17523	-37.9 (-49.7 to -26.0)	<0.001	16273	-43.6 (-55.8 to -31.5)	<0.001	16273	-5.6 (23.6 to 12.4)	0.54	15829	-7.4 (-19.6 to 4.8)	0.24
Women who did not change address	15191	-37.4 (-50.2 to -24.7)	<0.001	14196	-42.7 (-55.7 to -29.6)	<0.001	14196	-7.0(-26.3 to 12.3)	0.48	13818	-4.7 (17.8 yo 8.4)	0.48
LMP based GA only	16805	-35.4 (-47.5 to -23.2)	<0.001	15618	-408 (-53.3 to -28.4)	<0.001	15618	-3.2 (-21.6 to 15.1)	0.73	15195	-5.8 (-18.3 to 6.7)	0.36
Stratified analysis												
Oslo	4669	75 (-27.7 to 42.7)	0.68	4380	-5.9 (-42.8 to 31.0)	0.75				4285	12.5 (-24.3 to 49.3)	0.51
Akerhus	7547	10.5 (-22.8 to 43.9)	0.54	6982	8.9 (-25.4 to 43.1)	0.61				6759	29.2 (-4.8 to 63.1)	0.09
Bergen	3866	-15.6 (-43.7 to 12.4)	0.28	3577	-4.8 (-33.0 to 23.4)	0.74				3490	19.8 (-7.7 to 47.2)	0.16
Hordaland	1441	-37.6 (-104.6 to 29.4)	0.27	1334	-36.0 (-103.5 to 31.5)	0.30				1295	-26.7 (-92.7 to 39.2)	0.43
Not smoking	15440	-41.3 (-53.8 to -28.8)	<0.001	15229	-43.3 (-55.8 to -30.8)	<0.001	15229	-6.6 (-25.1 to 12.0)	0.49	14835	-5.6 (-18.2 to 6.9)	0.38
Smoking	1083	-28.3 (-80.0 to 23.3)	0.28	1044	-45.5 (-97.7 to 6.8)	0.09	1044	22.1 (-51.8 to 96.1)	0.56	994	-27.3 (-80.1 to 25.5)	0.31
Parity 0	8304	-16.8 (-33.3 to -0.4)	0.045	7803	-17.8 (-34.7 to -10)	0.04	7803	4.3 (-20.5 to 29.0)	0.74	7594	-8.3 (25.2 to 8.5)	0.33
Parity 1	6326	-0.6 (-20.6 to 19.4)	0.95	5858	-6.9 (-27.4 to 13.5)	0.51	5858	21.8 (-8.2 to 51.8)	0.15	5695	2.0(-18.3 to 22.4)	0.85
Parity ≥2	2893	-26.5 (-60.3 to 7.4)	0.13	2612	-31.0 (-66.4 to 4.4)	0.09	2612	17.8 (-31.7 to 67.4)	0.48	2540	-24.8 (-59.9 to 10.4)	0.17
Boys	8921	-30.7 (-47.5 to -13.8)	<0.001	8290	-39.6 (-57.0 to -22.2)	<0.001	<8290	-7.5 (-33.0 to 18.1)	0.57	8040	-5.4 (-22.8 to 12.1)	0.55
Girls	8602	-45.5 (-62. 0 to -29.1)	<0.001	7983	-47.8 (-64.8 to -30.8)	<0.001	7983	-3.6 (-28.9 to 21.8)	0.78	7789	-9.4(-26.4 to 7.6)	0.28
Education less than high school	985	-35.4 (-95.3 to 24.5)	0.25	968	-24.5 (-83.4 to 34.5)	0.42	968	-18.4 (-96 to 60.0)	0.65	905	-27.8 (-87.2 to 31.5)	0.36
Education high school	4173	-31.9 (-58.5 to 5.3)	0.02	4098	-36.0 (-62.3 to 9.7)	0.007	4098	10.4 (27.3 to 48.1)	0.59	3948	4.8 (-21.7 to 31.3)	0.72
Education up to 4 years of college	6474	-41.4 (-61.5 to -23.3)	<0.001	6403	-44.0 (-62.8 to -25.3)	<0.001	6403	-1.5 (-30.2 to 27.1)	092	6262	-4.9 (-23.7 to 13.9)	0.61
Educatiom more than 4 years of college (master of professional degree)	4866	-48.2 (-69.6 to 26.9)	<0.001	4804	-50.2 (-71.4 to -29.0)	<01001	4804	-17.8 (-49.4 to 13.8)	0.27	4714	-13.3 (-34.5 to 8.0)	0.22
Born in winter	4097	-20.2 (-46.6 to 6.2)	0.13	3797	-35.3 (-62.5 to 8.2)	0.01	3797	7.8 (-31.1 to 46.7)	0.69	3677	4.9 (-22.4 to 32.1)	0.73
Born in winter	4097	-20.2 (-46.6 to 6.2)	0.13	3797	-35.5 (-62.5 to -8.2)	0.01	3797	7.8 (-31.1 to 46.7)	0.69	3677	4.9 (-22.4 to 32.1)	0.73
Born in spring	4684	-60.6 (-82.2 to -39.0)	<0.001	4355	-60.2 (-82.2 to -38.3)	<0.001	4355	-46.7 ((79.5 to -13.8)	0.005	4226	-28.5(-50.6 to -6.4)	0.01
Born in summer	4626	-35.1 (-57.4 to -12.8)	0.002	4272	-40.5 (-63.3 to -17.6)	0.001	4272	14.2 (-20.7 to 49.1)	0.43	4167	-2.7 (-25.7 to 20.3)	0.82
Born in autumn	4116	-28.8 (-54.9 to -2.7)	0.03	3849	-31.9 (-58.6 to -5.3)	0.03	3849	16.1 (-23.0 to 55.1)	0.42	3759	5.1 (-21.4 to 31.7)	0.70

Effect estimate in grams per 10μg/m^3^ NO_2_

GA gestational age, LMP last menstrual period

^a^Model 1 adjusted for: maternal education, birth season, sex of child, maternal age, maternal marital status, maternal smoking during pregnancy, maternal height

^b^Model 2 adjusted for: maternal education, birth season, sex of child, maternal age, maternal marital status, maternal smoking during pregnancy, maternal height, area

^c^Model 3 adjusted for: maternal education, birth season, sex of child, maternal age, maternal marital status, maternal smoking during pregnancy, maternal height, parity, maternal weight, in stratified analysis the corresponding stratification variable is not included in the adjusment

However, reading across the columns of the table gives a different story. The successive sets of columns include adjustment for increasing numbers of factors that might affect the association. While model 1 still indicates a negative association between NO_2_ and birth weight that is highly significant (*p* < 0.001), models 2 and 3 do not. Inclusion of adjustment for parity or area and maternal weight has reduced the association such that the Betas have shrunk in magnitude to be closer to 0, with 95% CIs including 0 and *p*-values >0.05.

The table has multiple rows, with each one providing information on a different subset of the data, so the numbers in the analyses are all smaller than in the first row. The second row restricts the analysis to women who did not move address during pregnancy, an important consideration in estimating NO_2_ exposure from home addresses. The third row restricts the analysis to those whose gestational age was based on the last menstrual period. These second two rows present ‘sensitivity analyses’, performed to check that the results were not due to potential biases resulting from women moving house or having uncertain gestational ages. The remaining rows in the table present stratified analyses, with results given for each category of various variables of interest, namely geographical area, maternal smoking, parity, baby’s sex, mother’s educational level and season of birth. Only one row of this table has a statistically significant result for models 2 and 3, namely babies born in spring, but this finding is not discussed in the paper. Note the gap in the table in the model 2 column as it is not possible to adjust for area (one of the adjustment factors in model 2) when the analysis is being presented for each area separately.

### Tables of results – binary outcomes

Table 8 presents results from a study assessing whether children’s eating styles are associated with having a waist-hip ratio greater or equal to 0.5 (the latter being the outcome variable expressed in binary form – ≥0.5 versus <0.5) [17]. Results for boys and girls are presented separately, along with the number of children in each of the eating style categories. The main results are presented as crude and adjusted odds ratios (ORs). The adjusted ORs take account of age, exercise, skipping breakfast and having a snack after dinner, all of these being variables thought to affect the association between eating style and waist-hip ratio. Looking at the crude OR column, the value of 2.04 in the first row indicates that, among boys, those who report eating quickly have around twice the odds of having a high waist-hip ratio than those who do not eat quickly (not eating quickly is the baseline category, with an odds ratio given as 1.00). The 95% CI for the crude OR for eating quickly is 1.31 – 3.18. This interval does not include 1, indicating that the elevated OR for eating quickly is unlikely to be a chance finding and that there is a 95% probability that the range of 1.31 – 3.18 includes the true OR. The *p*-value is 0.002, considerably smaller than 0.05, indicating that this finding is ‘statistically significant’. The other ORs can be considered in the same way, but note that, for both boys and girls, the ORs for eating until full are greater than 1 but their 95% CIs include 1 and the *p-*values are considerably greater than 0.05, so not ‘statistically significant’, indicating chance findings.

**Table 8:** Results table from a study assessing whether children’s eating styles are associated with having a waist-hip ratio ≥0.5 or not [17]. Crude and adjusted odds ratios of eating quickly or eating until full for waist-to-height ratio (WHtr) ≥ 0.5

Variables	Total	WHtR ≥ 0.5	Crude		Adjusted	
	N	n (%)	OR (95% CI)	*P*-value	OR (95% CI)	*P*-value
Boys						
Eating quickly						
Yes	255	37 (14.5)	2.04 (1.31-3.18)	0.002	2.05 (1.31-3.23)	0.002
No	715	55 (7.7)	1.00		1.00	
Eating until full						
Yes	515	54 (10.5)	1.29 (0.83-1.99)	0.259	1.25 (0.80-1.95)	0.321
No	455	38 (8.4)	1.00		1.00	
Girls						
Eating quickly						
Yes	126	16 (12.7)	2.02(1.12-3.64)	0.020	2.09(1.15-3.81)	0.016
No	832	56 (6.7)	1.00		1.00	
Eating until full						
Yes	517	40 (7.7)	1.07 (0.66-1.74)	0.779	1.12 (068-1.82)	0.662
No	441	32 (7.3)	1.00		1.00	

*OR* odds ratio; *CI* confidence interval

Adjusted for age, exercise, skipping breakfast, and snack after dinner

The final columns present the ORs after adjustment for various additional factors, along with their 95% CIs and *p*-values. The ORs given here differ little from the crude ORs in the table, indicating that the adjustment has not had much effect, so the conclusions from examining the crude ORs are unaltered. It thus appears that eating quickly is strongly associated with a greater waist-hip ratio, but that eating until full is not.

## Conclusion

Summary tables of characteristics describe the study population and set the study in context. The main findings can be presented in different ways and choice of presentation is determined by the nature of the variables under study. Scrutiny of tables allows the reader to acquire much more information about the study and a richer insight than if the text only is examined. Constructing clear tables that communicate the nature of the study population and the key results is important in the preparation of papers; good tables can assist the reader enormously as well as increasing the chance of the paper being published.

## Abbreviations

ALRI: Acute lower respiratory infections
CI: Confidence interval
MUAC: Mid-upper-arm circumference
IQR: Inter-quartile range
NO_2_: Nitrogen dioxide
OR: Odds ratio
Q1: Quartile 1 (25th percentile)
Q2: Quartile 2 (50th percentile = median)
Q3: Quartile 3 (75th percentile)
SD: Standard deviation
